# Analysis of the Emotional Dynamics Associated with the Affective, Cognitive, and Behavioral Dimensions of Empathy among Adolescent Bystanders of Bullying Situations in Physical Education Classes

**DOI:** 10.5334/pb.1479

**Published:** 2026-07-01

**Authors:** Aurélien Besseling, Aurélie Wagener, Marc Cloes, Maurine Remacle, Alexandre Mouton, Elena Gemoets, Céline Stassart

**Affiliations:** 1 Department of Physical Activity and Rehabilitation Sciences, University of Liège, Building B21, 4 Allée des Sports, 4000 Liège, Belgium; 2 Research Unit for a life-Course perspective on Health & Education (RUCHE), University of Liège, Belgium; 3 Department of Psychology, University of Liège, Building B33, 1 Place des Orateurs, 4000 Liège, Belgium; 4 Centre de Formation des Enseignants (CEFEN), University of Liège, Building B32, 2 Place des Orateurs, 4000 Liège, Belgium

**Keywords:** Empathy, Bystanders, Bullying, Physical education, Young adolescents

## Abstract

Bullying represents a major issue in physical education (PE) contexts. The role of bystanders is crucial, with their empathy being an essential lever to react to such situations. However, this empathy does not always seem to be a sufficient condition for action. This study examines highly empathic students, who are more likely to engage in helping behaviors, in order to analyze the emotions related to the different dimensions of empathy as well as the factors that facilitate or hinder bystander helping behaviors toward victims in the context of PE. The study is based on a qualitative approach involving interviews with 20 students aged 12 to 14. Each interview consists of recalling a critical incident of bullying observed during PE classes, as well as two open-ended questions on factors influencing bystander intervention in this context. The findings indicate that emotions related to affective empathy can influence the type of response adopted. They also reveal that the emotional vocabulary used to describe victims’ emotions is still developing, and that early adolescence represents a particularly favorable period for its enrichment. Moreover, although students may display high levels of empathy, certain characteristics of PE classes could nevertheless generate fear, thereby hindering empathic processes and limiting their translation into helping behaviors. This research provides a better understanding of the relationships between empathy, the characteristics of PE classes, and reactions to bullying in this context, while offering directions for future research and a foundation for researchers to design interventions that can be implemented in PE classes.

## Introduction

### Bullying issue

Bullying is a major issue in the school environment. Olweus (1994) defines bullying behavior by three core characteristics: it is repetitive, deliberately harmful, and involves a power imbalance between the individuals involved. Although some researchers advocate revising this definition to incorporate cyberbullying, it has nonetheless underpinned numerous studies (Catheline, 2023) and continues to be cited as a key reference in recent research (Chu & Chen, 2025; Sjögren et al., 2025). Four forms of bullying are typically identified: physical (attacking, hitting, biting), verbal (insults, teasing), relational (gossiping, social exclusion), and cyberbullying (malicious or humiliating messages via electronic means) (Van Noorden et al., 2015). In the Wallonia-Brussels Federation (WBF), the Service for Health Information, Promotion, and Education (2023) uses Olweus’s definition to analyze bullying and reports that 20.8% of students in 7th and 8th grades (ages 12–14) have experienced it. This stage of schooling may be characterized by a temporary increase in victimization, linked to the disruptions and adjustments associated with the school transition (Vaillancourt et al., 2023). Such behaviors can have numerous negative consequences for victims, ranging from decreased academic performance and school refusal to the onset of anxiety, sleep and eating disorders, and even severe depressive episodes and suicidal thoughts (Catheline, 2020). While bullying can occur in various settings, it is particularly common in secluded areas where adult intervention is more limited such as toilets, quiet corners of the playground, hallways, or when teachers’ attention is elsewhere (Guilloux, Langbour & Debarbieux, 2024). The relationship between bullying and limited adult supervision highlights the importance of bystanders’ role, who are considered full-fledged actors, on the same level as the bully and the victim (Benmoyal Bouzaglo, 2022).

### Emotional dynamics underlying bystander intervention

In the literature, three types of bystander behaviors in response to bullying are identified: pro-bully, pro-victim, and passive (Benmoyal Bouzaglo, 2022). Research focuses on the factors that may influence helping behaviors among bystanders toward victims in bullying situations (Salmivalli, 2014). Such interventions are crucial, as bystanders’ behavior not only affects the likelihood that bullying will continue but also shapes the victim’s experience (Garandeau & Salmivalli, 2018). It is in this perspective that the emotional dynamics associated with bystander behavior have been studied, highlighting the central role of emotional information in this process. This involves both students’ own emotional states and their ability to recognize the emotions expressed by a person in distress (Trach & Hymel, 2020). How these emotions are perceived directly guides bystander behavior. From this perspective, empathy appears to be a central factor facilitating bystander intervention in bullying situations (Fredrick, Jenkins & Ray, 2020). Empathy can be defined as “*one’s capacities to resonate with another person’s emotions (affective empathy), understand his/her thoughts and feelings, distinguish one’s own thoughts and emotions from those of the observed (cognitive empathy), and respond with appropriate prosocial and helpful behavior (behavioral empathy)*” (Oliveira-Silva & Gonçalves, 2011, p. 201). This definition can be linked to the three key dimensions of Heyes’ (2018) model of empathy: affective, cognitive, and behavioral empathy. This model appears particularly relevant in the context of bystander intervention, as it includes an action-oriented dimension, namely the actual implementation of prosocial behavior.

#### Affective empathy

According to Heyes (2018), affective empathy is manifested through an emotional response automatically triggered by a stimulus, a phenomenon often referred to as “*emotional contagion*”. This process is reflected in an emotional state isomorphic to that experienced by others, resulting in a direct correspondence between the emotions of the observer and those of the person being observed. Bensalah, Caillies, and Anduze (2016) present a broader perspective, suggesting that affective empathy can also be expressed as a correspondence between different emotions of the same emotional valence (positive or negative).

#### Cognitive empathy

Cognitive empathy refers to the ability to understand what another person is feeling or thinking by accurately identifying and interpreting their emotions, without necessarily sharing those emotions oneself (Bensalah et al., 2016; Heyes, 2018). There are multiple ways of conceptualizing cognitive empathy. Heyes (2018) explained that cognitive empathy functions as a regulatory process capable of modulating affective empathy by adjusting the intensity of emotional responses. This conception, which links cognitive empathy and affective empathy, can be related to other-focused attribution, that is, the ability to understand that a shared emotion stems from another person’s situation (“*I feel sad because she is being mocked by the other students*”). Other conceptions also exist, such as self-focused attribution, which refers to the ability to imagine what one would feel in another person’s place (“*If I were in her place, I would feel sad too*”). Finally, some approaches draw a clearer distinction between cognitive and affective empathy by defining cognitive empathy as the ability to recognize or identify the emotions expressed by others (Bensalah et al., 2016). Cognitive empathy can also guide subsequent behavior, leading to controlled actions that may be prosocial or antisocial, referred to as behavioral empathy. From this perspective, the influence of cognitive empathy and even affective empathy on bystander intervention has been extensively examined, primarily through quantitative research (Fredrick et al., 2020; Lambe et al., 2019).

#### Behavioral empathy

Behavioral empathy refers to controlled responses that, when prosocial, aim to provide support or comfort to the victim (Heyes, 2018). These actions can be classified along two dimensions: the immediacy of the intervention, which corresponds to the timing of the action, and the level of involvement, which reflects the degree to which the individual immerse themselves in the incident (Bowes-Sperry & O’Leary-Kelly, 2005). These two dimensions give rise to four categories of behavior:

- High-immediacy, high-involvement behaviors (HH): the observer directly confronts the bully to stop the behavior;
- High-immediacy, low-involvement behaviors (HL): actions that interrupt the incident, such as distracting the harasser or removing the victim from the situation;
- Low-immediacy, high-involvement behaviors (LH): reporting the bully to a teacher.
- Low-immediacy, low-involvement behaviors (LL): discreet actions, such as comforting the target.

Finally, it should be noted that Bowes-Sperry and O’Leary-Kelly (2005) also consider non-intervention as a potential response (NR).

#### Other factors influencing empathic behavior

However, although the level of empathy plays an important role in bystander intervention, it does not fully account for helping behaviors. The model proposed by Latané and Darley (1970) suggests that bystanders go through a series of sequential stages before intervening: (1) noticing the event, (2) interpreting it as an emergency requiring assistance, (3) assuming responsibility to intervene, (4) knowing how to help, and (5) implementing the decision to act. This model highlights the importance of considering contextual influences (Jenkins & Nickerson, 2019).

### Emotional dynamics in the context of physical education

One of the relevant contexts of influence is physical education (PE). Unlike other school disciplines, which are more focused on developing cognitive skills, PE emphasizes the unity of body and mind (Messmer, 2018). Through sports activities, students can experience a wide variety of emotions (Cho, 2020) and face conflictual situations (Rico-Gonzalez, 2023). Furthermore, the emphasis on masculine ideals (e.g., strength, speed, and power), as well as the generally competitive nature of PE environments, shapes group norms and peer pressure (Borowiec et al., 2022). This reconfiguration of social norms and group status, specific to the PE context, may in turn influence bystanders’ emotions and represent an additional barrier to intervention (Mao & Yang, 2025). In addition to the masculine ideals they convey, PE classes also encourage students to expose their bodies to the gaze of others and to make their performance during activities visible (Webb, Quennerstedt & Öhman, 2008). Finally, the presence of multiple simultaneous activities in a large space makes supervision more complex and limits teachers’ ability to monitor all student behaviors (Cothran & Kulinna, 2014). This lack of supervision may generate concerns about personal safety, thereby constituting a major emotional barrier to intervention (Mao & Yang, 2025). These findings lead to the hypothesis that bystander intervention in PE may differ from that observed in other contexts. The literature further indicates that victims of bullying in secondary education report a significantly higher rate of bullying in gym and in locker rooms (Vaillancourt et al., 2010), highlighting the importance of better understanding bystander intervention in this specific context. To date, no study has directly examined the influence of emotion dynamics specific to PE on bystanders’ intervention in bullying situations in PE.

## Research Objectives

The aim of this study is to examine the expression of empathy among highly empathic bystanders confronted with bullying in PE. The originality of this study lies in the fact that most research assesses affective and cognitive empathy using self-report questionnaires, adopting a quantitative approach that does not fully capture the complexity of the emotions underlying empathy (Lambe et al., 2019; Simon & Nader-Grosbois, 2023). This study therefore seeks to go beyond empathy scores by adopting a qualitative approach focused on the emotions experienced and perceived as underlying empathy. This perspective, which remains underexplored in the literature, will help achieve a deeper understanding of emotional dynamics in PE. Research on bullying in this context remains limited (Wei & Graber, 2023), even though these emotional dynamics may potentially hinder intervention.

In addition to the study design, the originality of this research also lies in its sample. It has been shown that a high level of empathy does not necessarily lead to prosocial action, although it may act as a facilitating factor. In this context, this study focuses on students with high levels of empathy. This methodological choice is based on three main reasons. First, these students are more likely to take action and intervene to defend a victim, and therefore represent a population that warrants particular attention (Fredrick et al., 2020). Second, going beyond a simple empathy score by examining emotions, as well as the facilitators and barriers to helping behaviors within this population, may contribute to the development of strategies aimed at increasing the likelihood of bystander intervention. Finally, given that children’s understanding of emotion-related words has been identified as a predictor of empathy (Li & Yu, 2015), we expect to obtain rich and detailed data on emotional dimensions, thereby enhancing the depth and quality of the analyses. Our sample is also distinguished by the age of the participants, who are first-year secondary school students (aged 12–14 years), a developmental stage characterized by a temporary increase in victimization and a lower likelihood of helping a peer targeted by bullying (Jenkins & Nickerson, 2019; Vaillancourt et al., 2023).

This study, conducted with first-year secondary school students presenting a high level of empathy toward bullying situations in PE, is structured around three objectives:

- To analyze the emotions experienced by bystanders (affective empathy), the emotions they attribute to the victim (cognitive empathy), and how emotions influence their reactions (behavioral empathy);
- To identify the factors that facilitate or hinder bystander intervention in PE;
- To highlight the common characteristics of highly empathetic students who do not intervene in PE bullying situations.

## Methods

### Characteristics and design

The present study is part of a broader research project and is based on a previous study (Besseling et al., in press). The latter examined the prevalence of bullying in PE in WBF and explored the psychosocial factors (such as anxiety, empathy, peer relationships, assertiveness, and self-esteem) that foster prosocial behaviors among bystanders toward victims. That study was conducted with a convenience sample of 354 secondary school students. Their recruitment was carried out through emails and phone calls to the school administrations. Once the school administration gave its approval, consent was obtained from all individuals (students and their parents) involved in the study. The sample was designed to reflect the diversity of the students’ school locations, taking into account both the municipality and variation according to a socio-economic criterion. At this level, the only inclusion criterion was that participants were 7th-grade students in WBF.

The starting point of the present study was the full administration of the Basic Empathy Scale questionnaire (Jolliffe & Farrington, 2006). This scale consists of 20 items. Respondents rate each statement on a Likert scale from 1 (“*Strongly disagree*”) to 5 (“*Strongly agree*”). The total score ranges from 20 to 100 points and reflects the overall level of empathy, with two subscores: affective and cognitive empathy (respectively 11 and 9 items). The scale demonstrates good psychometric properties (e.g. Jolliffe & Farrington, 2006).

The present study adopts a qualitative methodological approach based on a cross-sectional design. It draws on a subsample of the initial group of 354 students, selected according to three criteria: parental consent to participate, empathy questionnaire scores, and participants’ gender. Of the 354 students, 89 (34 boys and 55 girls) obtained the necessary parental consent to participate in the interview. An analysis of their empathy scores was then conducted. One limitation of the empathy scale used is the absence of standardized criteria for classifying students as having low, medium, or high levels of empathy. To address this limitation, the average score obtained on the questionnaire was calculated across all 89 students, and those whose score exceeded 71.1, corresponding to the mean, were selected (Table 1). Based on this criterion, 46 students met the requirements to participate in the interviews. Girls and boys were contacted in descending order of their scores until 10 participants of each gender were reached, resulting in a total of 20 students. The intention to ensure a balance between girls and boys is explained by the fact that the literature highlights gender differences in empathy and prosocial behaviors (Garaigordobil, 2009; Jenkins & Nickerson, 2019). This finding led us to balance our sample in terms of the number of girls and boys, so that the responses would reflect both sexes regarding empathy and prosocial behaviors. The decision to limit the sample to 20 participants is explained by the exploratory nature of the study, whose main objective is to generate avenues for future analysis. Data saturation is generally reached after approximately 12 interviews (Guest, Bunce & Johnson, 2006). A psychologist conducted semi-structured interviews. The interviews were held online via the Teams application. Parents were asked to ensure that their adolescent was seated alone in a quiet, private room, free from noise and distractions, in order to maintain optimal conditions for the conversation. This study was approved by ethics committee of the faculty of psychology, speech therapy, and educational sciences of the University of Liège (file reference: 2324-036, amendment no. 2324-062). The personal data used in this study cannot be made available due to ethical constraints related to participant confidentiality.

**Table 1:** Empathy level by gender and sample size.


	EMPATHY *M* (*SD*)	AFFECTIVE EMPATHY *M* (*SD*)	COGNITIVE EMPATHY *M* (*SD*)

**Sample of 89 students**	71.1 (+/–12.5)	37.4 (+/– 7.9)	33.6 (+/– 5.7)

Boys (*n* = 34)	64.5 (+/– 10.8)	32.9 (+/– 6.9)	31.6 (+/– 5.6)

Girls (*n* = 55)	75.1 (+/– 11.8)	40.2 (+/– 7.3)	34.9 (+/– 5.3)

**Sample of 20 students**	77.7 (+/– 7.6)	41.4 (+/– 4.9)	36.3 (+/– 3.3)

Boys (*n* = 10)	74.1 (+/– 7.7)	38.9 (+/– 4.7)	35.2 (+/– 3.9)

Girls (*n* = 10)	81.4 (+/– 5.8)	43.9 (+/– 3.9)	37.5 (+/– 2.1)


### Participants

A group of 20 first-year secondary students, aged between 12 and 14 years, was formed, with a mean age of 12.3 years (+/– 0.47). The sample consisted of 10 boys and 10 girls, ensuring a balanced distribution by gender (Table 1). The students came from four different schools in WBF. Appendix A presents the characteristics of schools and students. It highlights the location of the schools, the socio-economic status of the municipalities in which they are located, as well as the distribution of boys and girls interviewed in each school. It also provides detailed information on each child, including age, gender, school, and empathy score.

Regarding the threshold, all the girls scored above the cut-off value of 71.1. In contrast, among the boys, four participants were included despite slightly lower scores: three scored 69 out of 100 and one scored 67 out of 100. The inclusion of these four boys is justified by two main reasons. The first relates to gender differences in empathy and prosocial behaviors identified in the literature (Garaigordobil, 2009; Jenkins & Nickerson, 2019). The second reason is that, although their scores are lower than the overall empathy mean of the 89 students (*M* = 71.1), they remain higher than the mean score observed among the 34 boys within the same sample (*M* = 64.5).

### Semi-structured interviews

The first part of the interview aims to address the first objective and analyzes empathy based on a bullying incident in PE observed by the student. In this regard, the starting point of this section is the description of this critical incident. If an adolescent was unable to report an incident, we proceeded directly to the second part of the interview. A critical incident can be defined as “*an event that may initially seem trivial but proves to be significant for the individual involved as well as for the people with whom they interact. This event usually takes place within a sensitive or delicate situation*” (Deslauriers, Deslauriers & LaFerrière-Simard, 2017). In the context of our research, this incident consisted of describing a recent situation in which the students had witnessed severe bullying of a classmate during a PE class. They were encouraged to give detailed answers to the questions: “*What happened? How did it happen? Who was involved? Where and when did it take place?*” *(Main question 1)*. No further details about the event were requested in order to limit the duration of the interview and prevent loss of attention. The choice of this method is justified by its ability to provide rich information, as critical incidents are almost always associated with emotions. Moreover, the decision to focus on a single incident is warranted by the exploratory nature of this approach and its still limited use in empathy research. As the critical incident served as the starting point, affective empathy was assessed by asking the participant, “*What emotions did you feel in response to this situation?*” *(Main question 2)*. Cognitive empathy was evaluated by focusing on the emotions of victims, using the question, “*What emotions did the victim feel?*” *(Main question 3)*. The student was systematically asked to describe the emotion in order to assess their understanding and the precision of their emotional vocabulary. To facilitate the expression of emotions, participants used the *Geneva Emotion Wheel* (Scherer, 2005), which was made accessible via screen sharing during the videoconference. This tool presents 20 distinct emotions, grouped into different emotional families and arranged around the perimeter of a wheel. Moreover, the options “*none*” and “*other*” allow the adolescent to indicate either the absence of emotion or an emotion not listed among the provided options. It is important to note that a single student could experience multiple emotions simultaneously. Finally, behavioral empathy is defined as the tendency to adopt different types of behaviors, including pro-aggressor, pro-victim, or passive responses (Benmoyal Bouzaglo, 2022). However, this study specifically focuses on the prosocial dimension of bystanders, and more precisely on pro-victim behaviors, i.e., actions intended to help the person in distress, as assessed through the question: “*How did you help the victim?*” *(Main question 4)*.

The second part aims to address the second objective and adopts a more open-ended approach. In contrast to the first part, which focused on a specific incident reported by the student, participants were invited to broaden their reflection beyond their personal experience by answering a question on intervention: “*In your opinion, what elements could facilitate helping behaviors toward victims of bullying during PE class?*” *(Main question 5)*, as well as a question on non-intervention: “*To your opinion, what could prevent someone from helping a bullying victim during PE class?*” *(Main question 6)*. The third objective is achieved through a cross-sectional analysis of the first and second parts of the interviews.

Overall, the interview included six main questions, some of which were accompanied by follow-up sub-questions, with the psychologist also having the opportunity to ask probing questions depending on participants’ responses. The interviews lasted an average of 25 minutes, ranging from 16 to 44 minutes depending on the adolescent’s talkativeness.

### Data analysis

#### Qualitative data analysis procedure

After the interviews were recorded, the responses from the 20 students were transcribed into a Word document using TurboScribe software. The transcripts were then carefully reviewed in order to correct any potential transcription errors.

For the first part of the interview, based on a critical incident, a deductive coding approach was used. This approach relied on metacodes and codes defined prior to the analysis. The metacodes corresponded to the four main questions in this section. Within each metacode, categories of responses corresponding to codes were created. Two coders participated in the inter-rater reliability analysis: the first, a doctoral student in motor sciences, and the second, a PhD holder specialized in PE didactics. This analysis was conducted in several steps. Once the code definitions were established, the first coder assigned each code to relevant verbatim excerpts from the transcripts. An Excel file was then created and sent to the second coder in order to assess coding reliability. Each sheet of the file corresponded to a metacode. Within each sheet, the first column contained the code definitions, the second column (hidden) contained the codes assigned by the first coder for each excerpt, the third column allowed the second coder to select a code via a drop-down list, and the final column contained the verbatim excerpts. The second coder’s task was to assign codes to the verbatim excerpts. Once completed, the file was returned to the first coder, who analyzed the level of agreement between both coders’ classifications. In cases of disagreement, code definitions and labels were revised, and the procedure was repeated until a satisfactory level of agreement was reached, confirming coding reliability. Two weeks after this step, the first coder also conducted an intra-rater reliability analysis by reapplying the coding procedure to all verbatim excerpts using the same Excel file.

The second part of the interviews, focusing on a broader exploration of bystanders’ helping behaviors toward victims, was analyzed using an inductive coding approach. The procedure used to assess inter- and intra-rater reliability was identical to that used in the deductive coding phase. The metacodes corresponded to the two main questions in this section. However, in contrast to the deductive approach, codes were generated from the interview data rather than being predefined.

All qualitative analyses were therefore subjected to reliability checks, yielding Cohen’s Kappa coefficients indicating an excellent level of agreement between raters, with inter-rater reliability above 0.80 and intra-rater reliability above 0.85 (Miles & Huberman, 1994).

Subsequently, several analyses were conducted in order to address the research objectives:

- The analysis of the impact of emotions related to bystanders’ affective and cognitive empathy on responses to a bullying situation in PE was conducted using contingency tables, allowing the distribution of felt and perceived emotions across different types of reactions to be examined;
- The analysis of factors facilitating or hindering bystander intervention in PE was carried out through an examination of the frequency of codes associated with facilitators and barriers;
- The analysis of common characteristics among students with high levels of empathy who did not intervene in a bullying situation was highlighted using a comparative table, including their levels of empathy, the characteristics of the critical incident, the emotions experienced, and the barriers to intervention.

#### Definition of codes derived from the deductive approach

The full set of metacodes and codes used in the deductive approach is presented in Table 2. In this section, the deductively defined codes are interpreted in light of the existing literature. Regarding bullying, they are based on the four forms proposed by Van Noorden et al. (2015): physical, verbal, relational, and cyberbullying. However, in the context of PE, the cyberbullying category was excluded and replaced by theft, as identified by Solberg and Olweus (2003) and not included in the classification of Van Noorden et al. (2015). It should be noted that a single incident could involve multiple forms of bullying.

**Table 2:** Presentation of metacodes and codes used in the deductive approach.


METACODES		CODES

Forms of bullying (*n* = 4)		Physical; Verbal; Relational; Theft

Affective empathy: emotions (*n* = 19)	Positive emotions (*n* = 9)	Admiration; Amusement; Contentment; Interest; Joy; Love; Pleasure; Pride; Relief

	Negative emotions (*n* = 10)	Anger; Contempt; Disappointment; Disgust; Fear; Guilt; Hatred; Regret; Sadness; Shame

Cognitive empathy: emotions (*n* = 20)	Positive emotions (*n* = 9)	Admiration; Amusement; Contentment; Interest; Joy; Love; Pleasure; Pride; Relief

	Negative emotions (*n* = 10)	Anger; Contempt; Disappointment; Disgust; Fear; Guilt; Hatred; Regret; Sadness; Shame

	Neutral emotion (*n* = 1)	Compassion

Behavioral empathy (*n* = 6)		LL; LH; HL; HH; NR; CMB


The codes related to affective empathy were based on the emotions from the Geneva Emotion Wheel, as well as certain combinations of emotions (Scherer, 2005). In this study, affective empathy is defined as a correspondence between different emotions of the same emotional valence (positive or negative) (Bensalah et al., 2016). This broader definition, which goes beyond a simple reproduction of another person’s emotional state, allows for a better understanding of the diversity of emotional responses in PE, which may shape the bystander’s reaction. To align with our definition of affective empathy, compassion was excluded from the analysis, as it does not have a clearly defined emotional valence (Scherer et al., 2013). Without compassion, the Geneva Emotion Wheel comprises 19 emotions. In the context of affective empathy, emotions for which no valence correspondence existed between the bystander and the victim were also excluded from the analysis. For example, when a student reported both positive and negative emotions but perceived only negative emotions in the victim, the positive emotions were excluded. The components excluded from the affective empathy analysis are, however, presented in the results section.

Different conceptions of cognitive empathy are proposed in the literature. Cognitive empathy can be related to affective empathy, particularly through other-focused attribution or self-focused attribution processes. It can also be considered independently of affective empathy, as the ability to recognize and identify emotions expressed by others (Bensalah et al., 2016). In the present study, we adopt a restricted definition of cognitive empathy, limited to the recognition and identification of others’ emotions. This choice is based on the sequential model of Latané and Darley (1970), according to which each stage of the bystander intervention process depends on the previous one. We therefore focus on the first stage, noticing the event, without which the subsequent stages cannot occur (Fredrick et al., 2020). This stage refers to the ability to notice an event and pay attention to relevant cues in the victim’s behavior, and thus to identify the other person’s emotions (Nickerson et al., 2014). In this context, codes related to cognitive empathy, that is, the emotions identified in the victim, were established based on the full range of emotions from the Geneva Emotion Wheel, as well as certain combinations of emotions (Scherer, 2005). The six forms of reactions associated with behavioral empathy are based on the classification proposed by Bowes-Sperry and O’Leary-Kelly (2005), who distinguish five types of behavior: LL, LH, HL, HH, and NR. An additional category was added: combination of multiple reactions (CMB).

## Results

The results will be organized into four sections, directly aligned with the study’s objectives. These results, based on a sample of 20 students, highlight trends but do not allow for generalization.

### Descriptive presentation of results related to critical incidents

**Figure 1:**
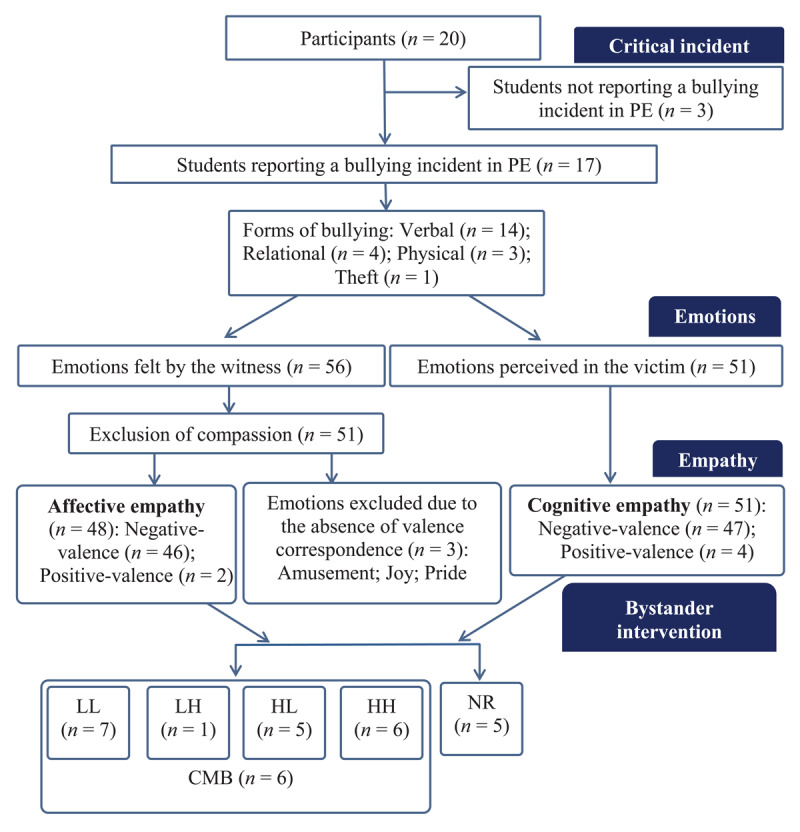
Schematic overview of the findings from the analysis of empathy in a critical incident.

Appendices A and B respectively provide more detailed information on each student (Appendix A) and on the characteristics of their responses (Appendix B). Figure 1 provides a schematic summary of the findings from the analysis of empathy in a critical incident and shows that 17 out of the 20 adolescents (85%) interviewed were able to describe a critical incident they observed in one of their PE classes. Among the forms of bullying mentioned by the students, verbal bullying was the most frequently reported. The majority of the teasing targeted students’ physical appearance and/or body odor. As an example, S1 (student 1) recounted a case concerning a classmate: “*He gained weight, and now many people make fun of him*.” Regarding relational bullying, it is especially apparent in team selection. Physical bullying is also reported and can take different forms. It appears as hitting for S7, striking for S10, and fighting for S14. In this study, none of the situations analyzed refer to cyberbullying. Finally, it is important to note that multiple forms of bullying can coexist within a single incident.

Figure 1 also illustrates the number of emotions experienced by witnesses in response to a bullying situation in PE, as well as the number of emotions perceived in the victim. To remain consistent with the definition of affective empathy found in the literature, compassion that was reported by five students was excluded from the analysis. The witnesses provided various justifications to explain their choice of compassion. For instance, S5 explained this emotion by putting herself in the other person’s shoes: “*Well, if I were in her place, I would have been sad because it’s not very nice to be picked last*.” The filtering conducted for affective empathy, based on the correspondence of emotional valence between the observer and the target, highlighted the exclusion of three positive emotions. S3 reported feeling positive emotions while the victims experienced only negative ones. She felt pride: “*For those who are chosen at the beginning, it’s still a bit of pride*” as well as joy as she added: “*Not being chosen last means that I have abilities*.” On the other hand, S14 explained that she felt amusement without knowing why: “*I don’t know how to explain why I laughed*.” Following this filtering, the analysis of affective empathy could be conducted. Considering the match in emotional valence between the observer and the target, 48 emotions were retained for affective empathy, compared to 24 when requiring an exact match between the experienced and perceived emotions (e.g., both the observer and the victim feel sadness). Of the 48 emotions reported by the students, 46 were of negative valence (95.8 %) and 2 were of positive valence (4.2 %). Regarding positive emotions, S16 explained that she felt relieved because “*the teachers intervened* ” and also perceived this relief in the victim: “*She was relieved that there were people coming to help her*.” For affective empathy, the most frequent emotions are sadness, anger, disgust, and disappointment.

For cognitive empathy, 47 of the 51 emotions were of negative valence (92.2 %), compared to four of positive valence (7.8 %). Of these 51 reported emotions, fear, anger, and sadness represented the majority, accounting for 56.9% (29/51).

Finally, among the four types of bystander reactions, three (LL, HL, HH) are reported fairly uniformly, whereas LH behaviors, such as reporting the bully to a teacher, occur less frequently. The number of students with no reaction (NR) and those exhibiting a combination of multiple reactions (CMB) is similar.

### Analysis of how affective and cognitive empathy influence bystanders’ reactions

This section highlights the most relevant results, while the full contingency tables are provided in Appendix C. Table 3 shows the distribution of students who experienced anger, disappointment, fear, and sadness (affective empathy) across different types of bystander reactions. The results show that, among students exhibiting HL behaviors, four out of five experience anger. S16 explains that she felt anger in particular: “*I was angry because there was no one else who stepped in. The boys didn’t really care; they were just watching, and then they were talking among themselves and everything*.” Among students experiencing anger, a larger proportion is found in the group combining multiple reactions than in the non-responding group. Among students who did not respond, the most frequently experienced emotions are sadness and disappointment. S13 did not react, explaining his disappointment: “*It was someone I got along with really well, and I honestly didn’t think they would want to do something like that*.” S2, who also did not intervene, explained that she felt a sense of sadness: “*Because I put myself in the person’s shoes and in their mind. They must have been sad about it. So, I kind of felt it as well for them*.” Conversely, the highest proportion of students combining multiple reactions also experienced sadness. S12, who adopted several types of reactions, explains: “*He is really nice, and what is happening to him isn’t cool. So, I feel a bit of sadness*.” Still among the students who remained passive, one in five experienced fear. According to S4, who did not respond and felt fear, his reaction is explained by the fact that “*it could happen to me too*.” These statements are also echoed by S11, who nevertheless intervened, and who explains: “*I knew that if I helped him, he would very clearly turn against me. So, I was afraid*.”

**Table 3:** Distribution of students who experienced anger, disappointment, fear, and sadness (affective empathy) according to the different types of bystander reactions.


	LL (*n* = 7)	LH (*n* = 1)	HL (*n* = 5)	HH (*n* = 6)	NR (*n* = 5)	CMB (*n* = 6)

Anger (*n* = 6)	3	0	4	2	1	3

Disappointment (*n* = 6)	2	0	3	0	3	2

Fear (*n* = 3)	2	0	0	1	1	1

Sadness (*n* = 10)	4	1	3	2	4	4


Table 4 presents the distribution of students who perceived emotions of anger, fear, sadness, and shame in the victim (cognitive empathy), across different types of bystander reactions. Among students exhibiting HH-type behaviors, more than half perceive anger in the victim. S16, who adopted an HH behavior, explains anger as follows: “*She was probably angry that her physical appearance was being targeted*.” Conversely, individuals perceiving fear in the victim are mostly found among those exhibiting LL-type behaviors. Regarding fear, S13 explains that he perceives it in the victim because the person is afraid “*that it will happen again*” while S16 states that the victim is afraid “*that they will go further*.” Regarding fear and shame, the proportion of individuals perceiving these emotions is lower among those who do not respond than among those combining multiple forms of intervention. S3, who adopted several forms of reactions, explains having perceived shame in the victim: “*Shame, because he must have been ashamed of not being chosen. It would make me feel a bit uncomfortable*.” Finally, the combination of two emotions does not reveal any new trends and remains consistent with those observed for each emotion considered separately.

**Table 4:** Distribution of students who perceived anger, fear, sadness, and shame in the victim (cognitive empathy) according to the different types of bystander reactions.


	LL (*n* = 7)	LH (*n* = 1)	HL (*n* = 5)	HH (*n* = 6)	NR (*n* = 5)	CMB (*n* = 6)

Anger (*n* = 7)	3	0	1	4	2	2

Fear (*n* = 8)	5	0	3	3	2	4

Sadness (*n* = 14)	6	1	4	5	5	6

Shame (*n* = 7)	4	1	3	2	1	4


### Identification of factors facilitating or hindering bystander intervention in physical education

This section aims to examine the factors that may facilitate or hinder bystanders’ helping behaviors toward victims in the specific context of PE classes, in order to identify actionable levers for enhancing bystander intervention with a high level of empathy. It is structured around two questions: “*What elements could facilitate helping behaviors toward victims of bullying during PE class?*” (facilitators) and “*What could prevent someone from helping a bullying victim during PE class?*” (barriers).

**Figure 2:**
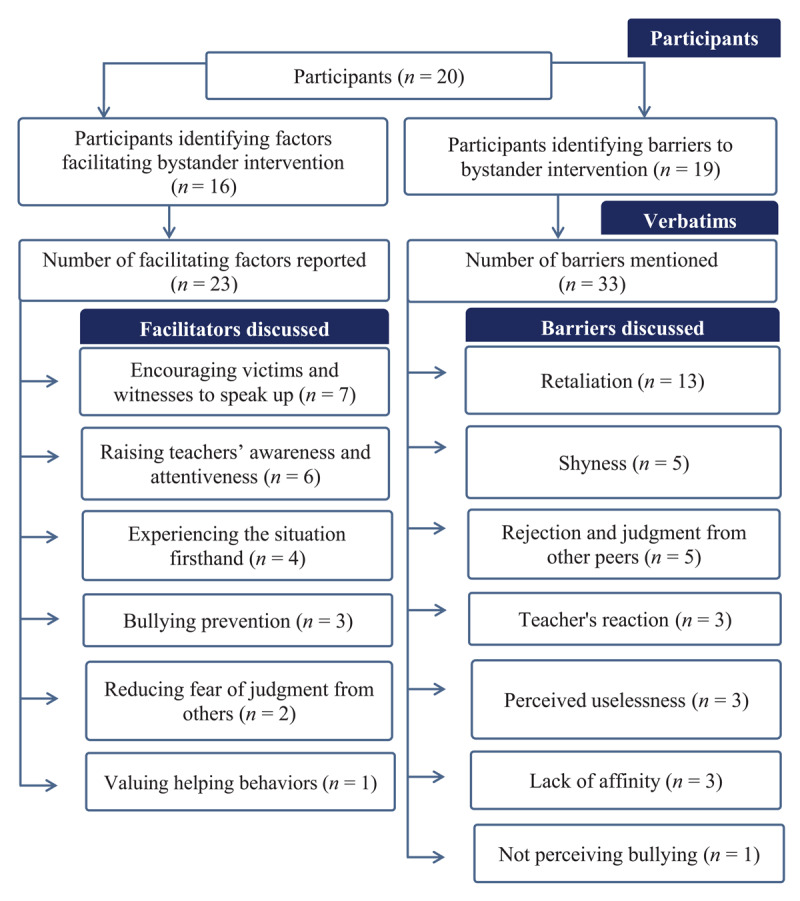
Schematic overview of codes and findings on facilitators and barriers to bystander intervention.

Figure 2 provides a schematic overview of the codes and findings related to facilitators and barriers to bystander intervention. Initially, 20 students were eligible to respond. Regarding facilitators, 16 students shared their views, producing 23 distinct verbatims, which were then grouped into categories. Among the 16 students, the number of verbatims provided ranged from 1 to 4 per person. For barriers, 19 students responded, generating 33 different verbatims, also classified into categories. Among the 19 students, each participant also provided between 1 and 4 verbatims.

Regarding facilitators, the most frequently mentioned were encouraging both victims and witnesses to speak up, as well as raising teachers’ awareness and attentiveness. S7 notably points out that “*there should be teachers serving as points of reference for bullying*.” while S1 suggests “*creating something like a kiosk* ” to encourage both witnesses and victims to speak up. S1 also emphasizes that it would be beneficial for “*the teacher to be more attentive*” since, according to S12, “*the teacher doesn’t care about the students*.” The third most commonly reported factor, having personally experienced a similar situation, is explained by S12: “*I had gone through something like that before and found it unfair what they were doing, that he was being mocked for it … So, I defended him*.” Other factors, such as bullying prevention, reducing fear of judgment from others, and valuing helping behaviors, are also mentioned. S11 notably highlights the importance of bullying prevention to understand “*the reactions we might have*.” S5, on the other hand, emphasizes the need to reduce fear, particularly so that people “*are not afraid of others’ judgment*.”

Among the barriers, retaliation emerges as the most significant factor, cited by more than half of them. This is followed by shyness as well as rejection and judgment from peers. To a lesser extent, the teacher’s reaction, perceived uselessness, and lack of affinity may also play a role, as does not perceiving the bullying. If the focus is on the verbatim statements themselves, out of the 33 identified verbatims, 16 (48.5 %) mentioned the term “*fear*”. This includes the fear of retaliation (“*Fear of being harassed too*” – S1); the fear of the teacher (“*Maybe fear of getting scolded by the teacher because we interrupted the class*” – S3); and the fear of rejection (“*It was more about not jeopardizing the relationship with the class and not getting too involved* ” – S5).

### Analysis of highly empathetic students who do not intervene in bullying situations in physical education

The third objective aims to highlight the common characteristics of highly empathetic students who do not intervene in bullying situations in PE classes. Among the 17 students who reported a critical incident, 5 did not react (S2, S4, S5, S13 and S14), despite showing high levels of empathy. Table 5 presents the shared characteristics of S2, S5 and S13. These three students obtained empathy scores above their gender-based subgroup mean. S2 and S5 scored above the girls’ subgroup average (*M* > 75.1; *n* = 55), while S13 also scored above the boys’ subgroup average (*M* > 64.5; *n* = 34). These students were exposed to verbal or relational bullying and all three reported mainly negative or neutral emotions, particularly compassion. A common barrier also emerges: fear. S2 expresses this through the “*fear of what they might say or do*.” Similarly, S13 explains his fear by stating “*that others might also turn against me*.” S5, in turn, is mainly characterized by fear of rejection. Students S4 and S14 present slightly different profiles. S4 highlights a perceived uselessness of intervention, stating that “*it wouldn’t have made much difference since he already had all his friends around him*”, as well as a lack of affinity: “*they are not my friends, we can’t force them to go to others to comfort them*.” S14, on the other hand, reports a positive emotion, namely amusement, explaining that she “*laughs because, well, I don’t know how to explain why I laughed*.”

**Table 5:** Characteristics of students S2, S5, and S13 in non-intervention situations following critical incidents of bullying in PE classes.


	S2	S5	S13

Empathy score	82	79	69

Forms of bullying	Verbal	Relational	Verbal

Experienced emotions	Anger; Compassion; Sadness; Disgust	Compassion; Shame; Disappointment	Sadness; Hatred; Disappointment

Barriers	Fear of retaliation	Fear of rejection	Shyness, Fear of retaliation


## Discussion

For reference, this research was guided by three objectives: to analyze bystanders’ emotions associated with affective and cognitive empathy and their influence on reactions (behavioral empathy); to identify factors that facilitate or hinder bystander intervention in PE; and to examine the common characteristics of highly empathetic students who do not intervene in PE bullying situations.

### Analysis of how affective and cognitive empathy influence bystanders’ reactions

The analysis of affective empathy suggests that experienced emotions are expressed at the behavioral level through various forms of responses (Moore, Gillanders & Stuart, 2022). Among the five students exhibiting HL behavior, four reported feeling anger. This observation can be linked to the concept of “*negative urgency*”, defined as the tendency to lose self-control in response to negative emotions (Chester et al., 2016). In this context, anger, by disrupting self-control, may lead to impulsive reactions, such as directly confronting the aggressor (Chester et al., 2016). However, anger is an ambivalent emotion: it can also motivate action and act as a driver of justice, which helps explain its role in bystanders’ responses to harassment situations (Hess, 2014). S16 highlights anger as a potential driver of justice when explaining: “*I was angry because there was no one else who stepped in. The boys didn’t really care; they were just watching, and then they were talking among themselves*.” Moreover, sadness was observed in similar proportions among students, whether they remained passive or combined different responses. This finding is consistent with the literature, which shows that sadness can lead to withdrawal, feelings of helplessness, and distress (Zaid et al., 2021), but can also promote more cautious, polite, and other-oriented interpersonal strategies (Forgas, 2017). The polite strategy is illustrated by S12, who experienced sadness and adopted two low-implication intervention strategies. He explains: “*We include him in our group and play with him*”, while also engaging in a measured confrontation with the aggressor: “*I first asked why they were doing this?*”, before adding that he “*mainly focuses on supporting the victim*.” The fact that emotions arising from affective empathy processes may shape the way individuals react highlights the importance of fostering students’ awareness of their emotions and the behaviors associated with them (Goleman, 2014).

Regarding the emotions identified in the victim through cognitive empathy processes, our study highlights the limited vocabulary used by students. Although the selection of this highly empathic sample was partly motivated by the assumption that these students might demonstrate stronger emotion word comprehension (Li & Yu, 2015), the present study suggests that students’ ability to understand others’ emotions (cognitive empathy) is still developing. This observation is reflected in the type of emotions identified, as 56.9% of the emotions attributed to the victim correspond to fear, anger, or sadness, which are basic emotions (Ekman, 1992). This illustrates that mastery of emotional vocabulary remains partial. Emotional vocabulary becomes more complex with age, and adolescents progressively adopt more precise and abstract terms (Grosse & Streubel, 2024). This period of adolescence appears particularly conducive to the acquisition of knowledge about emotions (Dylman, Blomqvist & Champoux-Larsson, 2020). Given that mastery of emotional vocabulary is a prerequisite for cognitive empathy (Li & Yu, 2015), it could be beneficial to develop students’ emotional repertoire, as well as their understanding of emotions through the interpretation of facial expressions, the identification of their triggers, and the assessment of their intensity.

### Identification of factors facilitating or hindering bystander intervention in physical education

The identification of factors facilitating or hindering bystander intervention in PE highlights that barriers to helping behaviors may be related to the characteristics of the PE class environment. This finding is reinforced by the fact that the most frequently cited barrier to bystander intervention is “fear of retaliation”, mentioned by 13 students. In light of this finding, it can be suggested that, given the specific context of PE classes, the perceived risk associated with intervening as a bystander may be greater. This sense of insecurity may stem from the organizational context of PE classes, which can limit teachers’ ability to monitor all behaviors (Cothran & Kulinna, 2014). This limitation becomes even more pronounced in locker rooms, which are spaces with restricted adult accessibility (Le Cloirec, 2020). It is also possible that the difficulties of intervening, linked to a lack of supervision in PE, are reflected in the facilitators of intervention, with six students highlighting the importance of “raising teachers’ awareness and attentiveness”. The issue of limited supervision is illustrated by S14, who explains: “*People are afraid to tell teachers*.” This statement suggests that certain incidents go unnoticed by teachers. It also points to another barrier identified by three students, namely the fear of the teacher’s reaction.

This barrier could be mitigated by the most frequently cited facilitator of intervention among students, namely “*encouraging victims and witnesses to speak up*”. In this respect, S1 proposes that it would be beneficial to “*a reporting station*”. In addition to its perceived insecurity, PE classes may be associated with masculine ideals of strength, speed, and power. Adolescents who conform to these norms often achieve higher social status (Borowiec et al., 2022). This power imbalance is highlighted by S2, who explains: “*Those who were making fun in our school were somewhat considered superior to others. As a result, we did not really try to oppose them*.” This context may contribute to fears of “*rejection and judgment from other peers*”, which were reported as a barrier by five students. Finally, PE classes may be associated with greater social exposure than in other subjects, particularly due to the visibility of bodies and performance (Webb et al., 2008), which could contribute to the emergence of an anxiety-inducing climate. This climate represents an emotional barrier, particularly associated with fear (Mao & Yang, 2025). Our findings may support this: among the 33 verbatim responses collected regarding barriers to intervention, 16 (48.5%) explicitly mention “*fear*”.

### Analysis of highly empathetic students who do not intervene in bullying situations in physical education

The analysis of the five students who did not react to a bullying situation in PE reveals similar profiles among three of them. These students demonstrate a high level of empathy and report experiencing strong negative emotions, such as anger, disgust, shame, or hatred. However, their decision to take action is mainly hindered by fear. This analysis suggests that PE classes, due to their specific characteristics, may evoke fear. However, it remains to be tested whether this effect is specific to PE or not. Therefore, further research is needed to investigate this hypothesis. This fear is not part of the emotional processes underlying affective empathy; however, it can disrupt these processes and hinder the expression of helping behaviors (i.e., behavioral empathy), even among highly empathetic students. In programs aimed at promoting bystander helping behaviors toward victims, the development of empathy represents a key lever (Fredrick et al., 2020). However, in classrooms characterized by heterogeneous levels of empathy, it also appears essential to reduce fear associated with intervention. This fear may indeed hinder empathic processes, particularly in contexts where engaging in helping behaviors could be perceived as anxiety-provoking, such as PE classes. Fear can be effectively addressed through cognitive-behavioral therapy (CBT), particularly via exposure therapy, which gradually confronts individuals with feared situations to reduce their fear (Rector, 2015). PE could utilize exposure therapy through the role of the referee: implemented gradually and within a safe environment, it would allow students to practice intervening while reducing their apprehension about potential repercussions. In addition to refereeing, the results offer several avenues for encouraging bystander intervention in cases of bullying in PE. In particular, teachers can help students become more aware of their emotions and better understand how these influence their behavior. They can also enrich students’ emotional vocabulary. It also appears important for teachers to show students that they are being listened to, in order to foster a safer emotional and relational climate in PE classes. From this perspective, designing a program that integrates all of these components appears to be a particularly relevant approach.

## Limitations

The limitations of our study concern both the theoretical framework and the selected sample. Heyes’ (2018) theoretical framework distinguishes three dimensions of empathy: affective, cognitive, and behavioral. The way this framework was applied in our research has certain limitations. In our study, the emotions considered for affective empathy were based on a valence match between felt and perceived emotions, in line with the broader perspective proposed by Bensalah et al. (2016). Heyes’ (2018) model, however, assumes a stricter emotional correspondence. Cognitive empathy was examined solely through the identification of others’ emotions, although this dimension includes additional components, making our analysis partial. The empathy scale used in this study measures the affective and cognitive dimensions but does not include the behavioral dimension. Moreover, the threshold was determined based on the mean of our sample, which does not allow us to know whether it would be the same in another sample. The sample also presents certain limitations. With only 20 students, the generalizability of the results is limited. Participants were asked to describe a recent situation, introducing a temporal factor that could lead to memory bias. Moreover, it would also have been useful to initially ask adolescents whether they had witnessed bullying situations. In our study, two girls and one boy were unable to describe any specific incident. Finally, to maintain a certain gender balance, four boys with a score below 71.1/100 on the Basic Empathy Scale were included in the sample.

## Perspectives

Based on the preliminary results of this study, it appears important to develop a deeper understanding of how the characteristics of PE classes influence the enactment of helping behaviors among bystanders. In this regard, it seems necessary to further explore the notion of fear. Future research could specifically examine the role of body exposure, performance demands, masculinity ideals associated with PE classes, as well as supervision challenges, in shaping this fear of intervening in the PE context. Moreover, the findings highlight the importance of moving beyond simple empathy scores in order to investigate underlying emotions. A more fine-grained analysis of individual and contextual characteristics is therefore needed to understand why an emotion may, depending on the situation, act either as a facilitator or as a barrier to helping behaviors. These considerations support the use of qualitative methodologies in the study of empathy, in order to better capture emotional subtleties and the mechanisms involved. These findings could also contribute to a better understanding of the key factors that should be considered when designing programs to prevent bullying in PE.

## Conclusion

The literature highlights that PE classes constitute a context conducive to bullying. It also emphasizes the central role of bystanders and their empathy in the development of helping behaviors toward victims. However, empathy alone does not appear to fully explain helping behaviors. This study therefore focuses on highly empathic students, who are more likely to intervene. The present study adopts a qualitative methodology by interviewing 20 students about a critical bullying incident they observed in PE, in order to analyze the emotions associated with the different dimensions of empathy. It also aims to highlight the facilitators and barriers to bystander intervention in this context. The results suggest that emotions associated with affective empathy can, in some cases, influence the type of reaction adopted. In addition, the emotional vocabulary used to describe victims’ emotions appears to still be developing, and early adolescence seems particularly well suited to fostering its development. It also appears that, among these highly empathic students, certain characteristics of PE classes can elicit fear, which interferes with empathic processes and limits the transition to helping behavior.

## Additional Files

The additional files for this article can be found as follows:

10.5334/pb.1479.s1 Appendix A. Characteristics of schools and students.

10.5334/pb.1479.s2 Appendix B. Analysis of each student’s response characteristics.

10.5334/pb.1479.s3 Appendix C. Distribution of emotions associated with affective and cognitive empathy across six forms of bystander intervention.
