# Supplementary material for: Analysis of the Emotional Dynamics Associated with the Affective, Cognitive, and Behavioral Dimensions of Empathy among Adolescent Bystanders of Bullying Situations in Physical Education Classes
Source: Psychol Belg. 2026 Jul 1;66(1):98–113. doi: 10.5334/pb.1479 (PMC13330851; doi:10.5334/pb.1479)
Supplement: Appendix A. — Characteristics of schools and students. [file pb-66-1-1479-s1.pdf]

Appendix A: Characteristics of schools and students

	Schools			
**School characteristics**	**School 1**	**School 2**	**School 3**	**School 4**
Province	Liège	Liège	Liège	Luxembourg
Mixed or single-sex PE classes	Single-sex	Single-sex	Single-sex	Single-sex
Average net taxable income per inhabitant in the municipality of the school (2023)	20 551€	23 928€	17 861€	21 547€
**Characteristics of interviewed students**	**School 1**	**School 2**	**School 3**	**School 4**
Mean age (years)	12.2	12	12.5	12.5
Number of boys interviewed	3	0	2	5
Number of girls interviewed	2	3	2	3
Average score on the empathy questionnaire (/100)	76.8	81	79	76.5

Characteristics of schools and students by school

N°	Age	Gender	Schools	Empathy questionnaire (/100)
1	13	Boy	School 1	75
2	12	Girl	School 1	82
3	12	Girl	School 2	88
4	12	Boy	School 1	67
5	13	Girl	School 4	79
6	13	Girl	School 4	74
7	12	Boy	School 1	72
8	12	Girl	School 2	75
9	12	Girl	School 2	80
10	13	Boy	School 3	73
11	13	Boy	School 4	87
12	13	Boy	School 4	89
13	12	Boy	School 4	69
14	12	Girl	School 3	87
15	14	Boy	School 4	71
16	13	Girl	School 3	87
17	12	Boy	School 4	69
18	12	Girl	School 4	74
19	12	Girl	School 1	88
20	12	Boy	School 3	69

Individual characteristics of each student
