# Supplementary material for: Analysis of the Emotional Dynamics Associated with the Affective, Cognitive, and Behavioral Dimensions of Empathy among Adolescent Bystanders of Bullying Situations in Physical Education Classes
Source: Psychol Belg. 2026 Jul 1;66(1):98–113. doi: 10.5334/pb.1479 (PMC13330851; doi:10.5334/pb.1479)
Supplement: Appendix B. — Analysis of each student’s response characteristics. [file pb-66-1-1479-s2.pdf]

N°	Critical incident		Number of emotions associated with affective empathy	Number of emotions associated with cognitive empathy	Behavioral response types associated with behavioral empathy	Number of facilitators mentioned	Number of barriers mentioned
	Sharing a critical incident	Forms of bullying					
1	Yes	Verbal	3	3	HH	2	1
2	Yes	Verbal	4	1	NR	1	1
3	Yes	Verbal, Relational	5	3	LH, HL	1	4
4	Yes	Theft	4	4	NR	1	4
5	Yes	Relational	3	3	NR	1	1
6	Yes	Verbal	6	1	HL	1	2
7	Yes	Physical, Verbal	1	4	LL, HH	1	1
8	Yes	Verbal	1	3	LL	1	1
9	No	/	/	/	/	0	0
10	Yes	Physical, Verbal, Relational	5	3	LL	2	1
11	Yes	Verbal, Relational	4	5	LL, HH	2	2
12	Yes	Verbal	3	3	LL, HL	2	2
13	Yes	Verbal	3	3	NR	0	2
14	Yes	Physical	2	2	NR	1	1
15	No	/	/	/	/	1	1
16	Yes	Verbal	4	5	LL, HL, HH	1	4
17	Yes	Verbal	2	2	HH	1	1
18	Yes	Verbal	3	3	LL, HL	4	2
19	No	/	/	/	/	0	1
20	Yes	Verbal	3	3	HH	0	1

Appendix B: Analysis of each student’s response characteristics
