# Supplementary material for: Analysis of the Emotional Dynamics Associated with the Affective, Cognitive, and Behavioral Dimensions of Empathy among Adolescent Bystanders of Bullying Situations in Physical Education Classes
Source: Psychol Belg. 2026 Jul 1;66(1):98–113. doi: 10.5334/pb.1479 (PMC13330851; doi:10.5334/pb.1479)
Supplement: Appendix C. — Distribution of emotions associated with affective and cognitive empathy across six forms of bystander intervention. [file pb-66-1-1479-s3.pdf]

Appendix C: Distribution of emotions associated with affective and cognitive empathy across six forms of bystander intervention

	**Bystander reactions to bullying – Behavioral empathy (number of reactions)**					
**Number of felt emotions in affective empathy (n=48)**	**LL (n=7)**	**LH (n=1)**	**HL (n=5)**	**HH (n=6)**	**NR (n=5)**	**CMB (n=6)**
Amusement (n=1)	0	0	0	1	0	0
Anger (n=6)	3	0	4	2	1	3
Contempt (n=2)	0	0	1	0	1	0
Disappointment (n=6)	2	0	3	0	3	2
Disgust (n=6)	3	0	2	3	1	2
Fear (n=3)	2	0	0	1	1	1
Guilt (n=4)	2	1	1	2	0	2
Hatred (n=1)	0	0	0	0	1	0
Regret (n=3)	1	0	1	1	0	0
Relief (n=1)	1	0	1	1	0	1
Sadness (n=10)	4	1	3	2	4	4
Shame (n=5)	1	1	2	1	1	1
Anger & Sadness (n=4)	2	0	2	1	1	2
Disappointment & Sadness (n=4)	2	0	2	0	2	2

	Bystander reactions to bullying - Behavioral empathy (number of reactions)					
**Number of perceived emotions in cognitive empathy (n = 51)**	**LL (n=7)**	**LH (n=1)**	**HL (n=5)**	**HH (n=6)**	**NR (n=5)**	**CMB (n=6)**
Amusement (n=2)	0	0	0	2	0	0
Anger (n=7)	3	0	1	4	2	2
Disappointment (n=3)	1	0	1	1	1	1
Disgust (n=2)	1	1	1	0	0	1
Fear (n=8)	5	0	3	3	2	4
Guilt (n=3)	2	0	1	2	1	2
Hatred (n=3)	2	0	0	1	1	1
Relief (n=2)	2	0	1	2	0	2
Sadness (n=14)	6	1	4	5	5	6
Shame (n=7)	4	1	3	2	1	4
Fear & Sadness (n=8)	5	0	3	3	2	4
Anger & Sadness (n=6)	2	0	1	4	2	2
Sadness & Shame (n=5)	3	1	3	1	1	4
